# Supplementary figures and images for: The identification of blood-derived response eQTLs reveals complex effects of regulatory variants on inflammatory and infectious disease risk
Source: PLoS Genet. 2025 Apr 10;21(4):e1011599. doi: 10.1371/journal.pgen.1011599 (PMC12013874; doi:10.1371/journal.pgen.1011599)

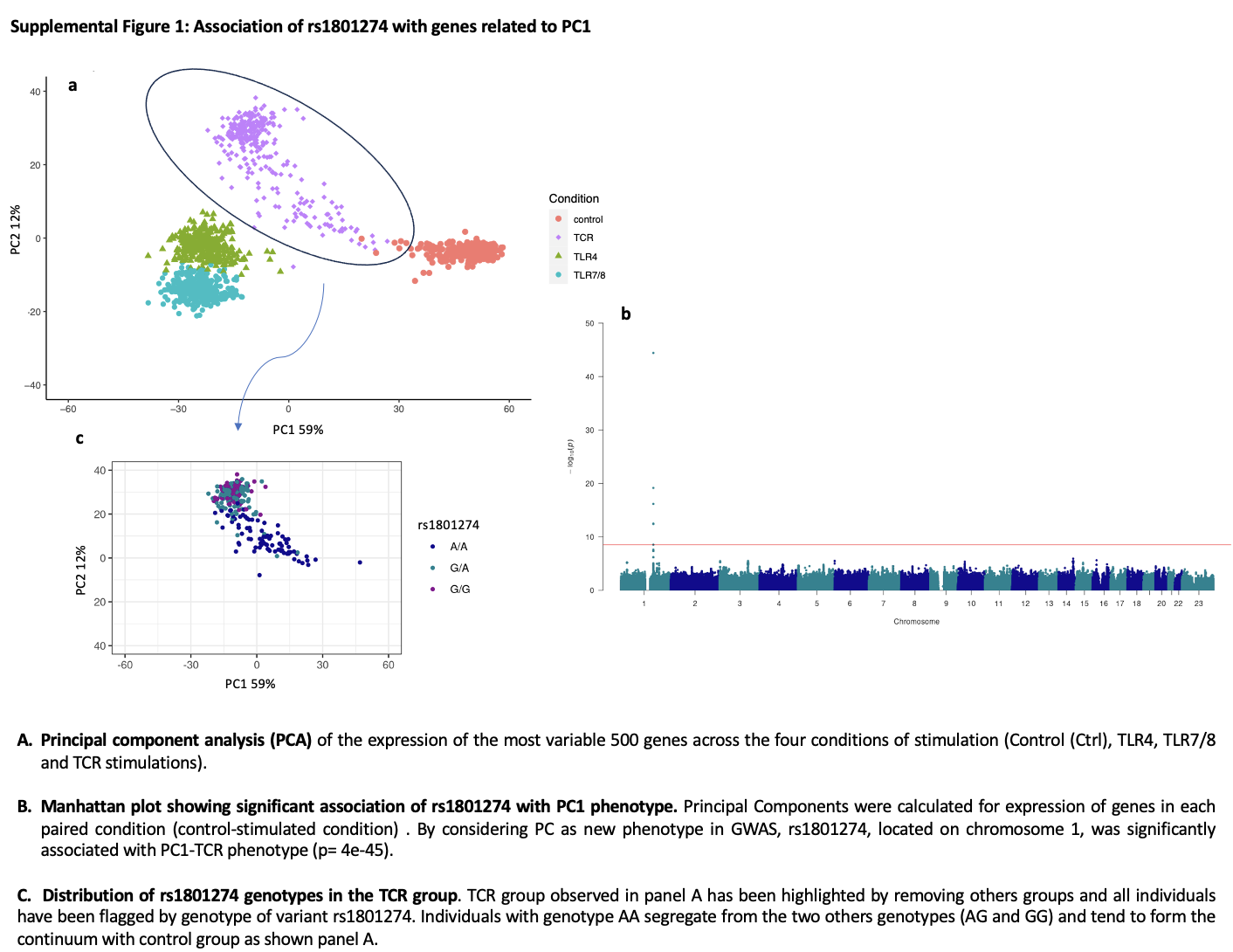

Supplement: S1 Fig — A. Principal component analysis (PCA) of the expression of the most variable 500 genes across the four conditions of stimulation (Control (Ctrl), TLR4, TLR7/8 and TCR stimulations). B. Manhattan plot showing significant association of rs1801274 with PC1 phenotype. Principal Components were calculated for expression of genes in each paired condition (control-stimulated condition). By considering PC as new phenotype in GWAS, rs1801274, located on chromosome 1, was significantly associated with PC1-TCR phenotype (p= 4e-45). C. Distribution of rs1801274 genotypes in the TCR group. TCR group observed in panel A has been highlighted by removing others groups and all individuals have been flagged by genotype of variant rs1801274. Individuals with genotype AA segregate from the two others genotypes (AG and GG) and tend to form the continuum with control group as shown panel A. (TIFF) [file pgen.1011599.s001.tiff]

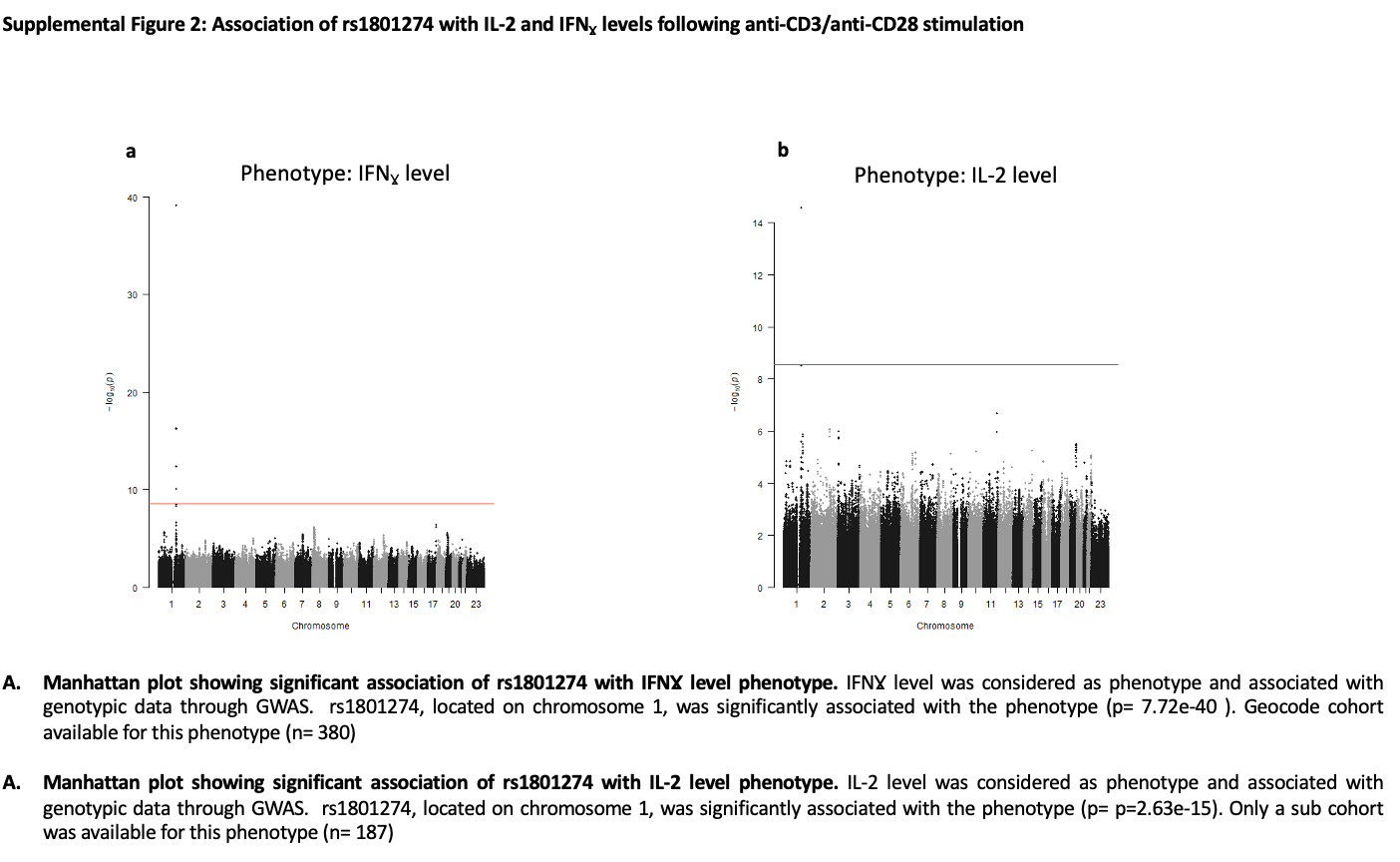

Supplement: S2 Fig — A. Manhattan plot showing significant association of rs1801274 with IFNƔ level phenotype. IFNƔ level was considered as phenotype and associated with genotypic data through GWAS. rs1801274, located on chromosome 1, was significantly associated with the phenotype (p= 7.72e-40). Geocode cohort available for this phenotype (n= 380). B. Manhattan plot showing significant association of rs1801274 with IL-2 level phenotype. IL-2 level was considered as phenotype and associated with genotypic data through GWAS. rs1801274, located on chromosome 1, was significantly associated with the phenotype (p= p=2.63e-15). Only a sub cohort was available for this phenotype (n= 187). (TIFF) [file pgen.1011599.s002.tiff]

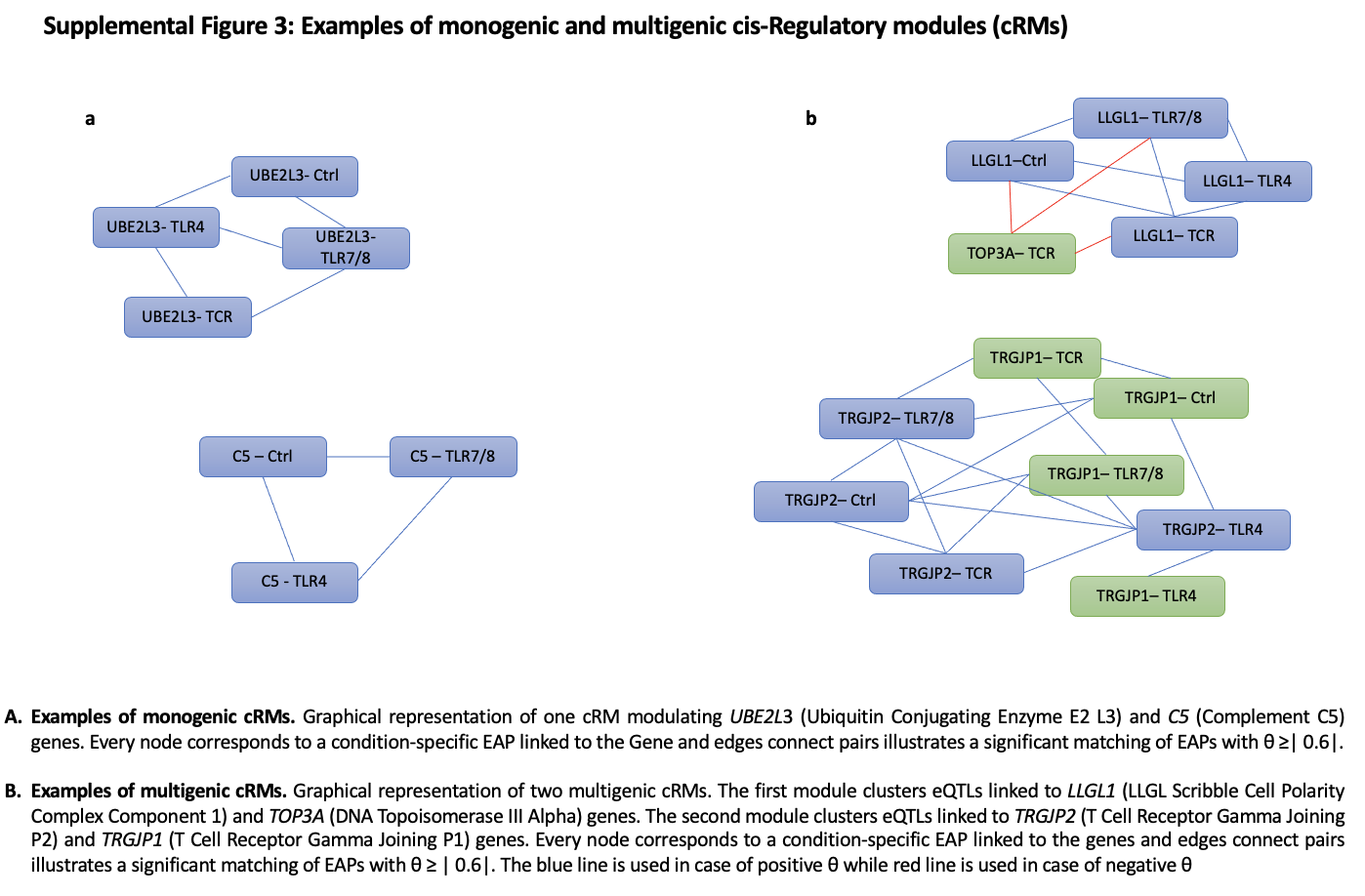

Supplement: S3 Fig — A. Examples of monogenic cRMs. Graphical representation of one cRM modulating UBE2L3 (Ubiquitin Conjugating Enzyme E2 L3) and C5 (Complement C5) genes. Every node corresponds to a condition-specific EAP linked to the Gene and edges connect pairs illustrates a significant matching of EAPs with θ ≥| 0.6|. B. Examples of multigenic cRMs. Graphical representation of two multigenic cRMs. The first module clusters eQTLs linked to LLGL1 (LLGL Scribble Cell Polarity Complex Component 1) and TOP3A (DNA Topoisomerase III Alpha) genes. The second module clusters eQTLs linked to TRGJP2 (T Cell Receptor Gamma Joining P2) and TRGJP1 (T Cell Receptor Gamma Joining P1) genes. Every node corresponds to a condition-specific EAP linked to the genes and edges connect pairs illustrates a significant matching of EAPs with θ ≥ | 0.6|. The blue line is used in case of positive θ while red line is used in case of negative θ. (TIFF) [file pgen.1011599.s003.tiff]

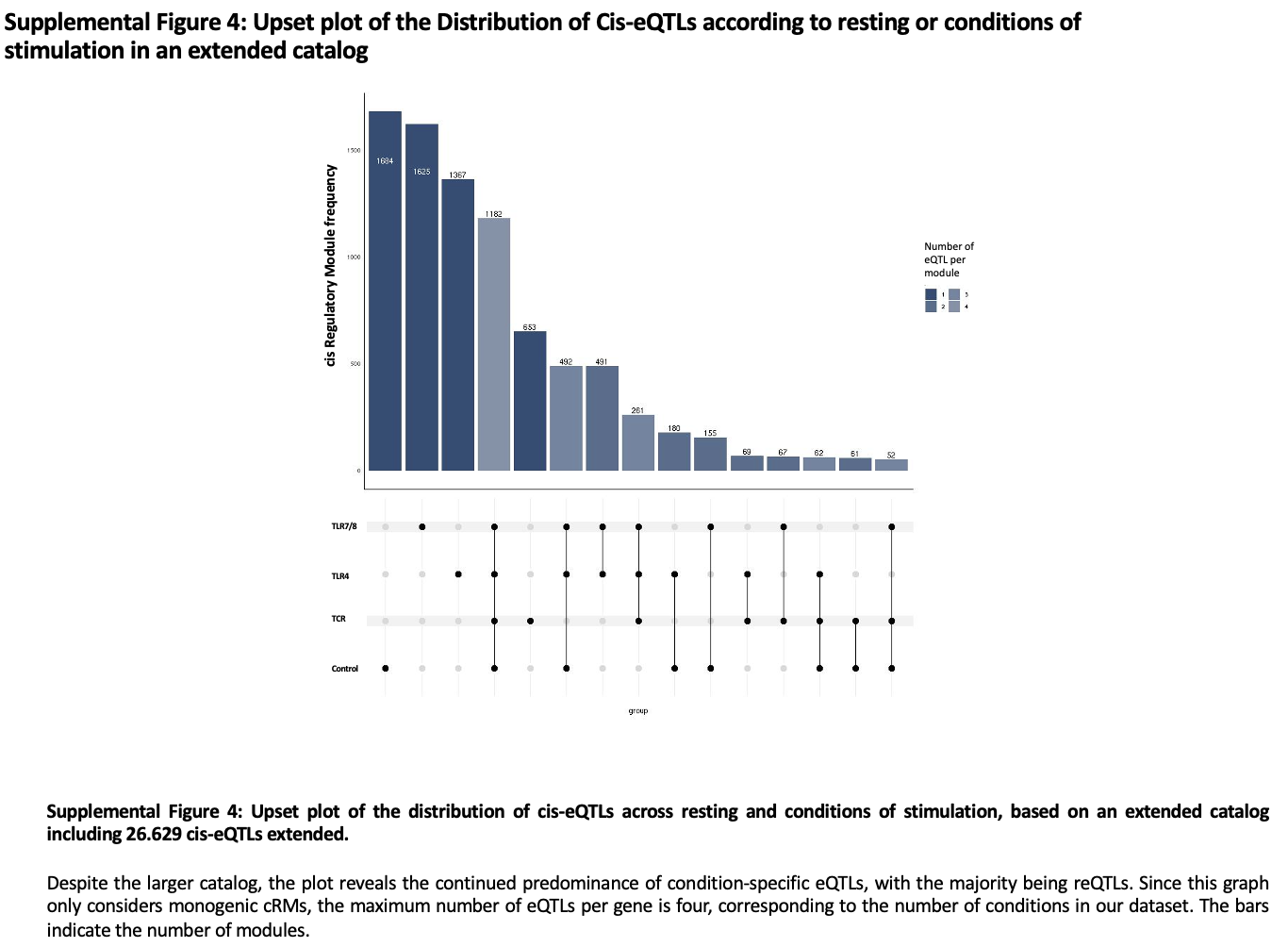

Supplement: S4 Fig — Despite the larger catalog, the plot reveals the continued predominance of condition-specific eQTLs, with the majority being reQTLs. Since this graph only considers monogenic cRMs, the maximum number of eQTLs per gene is four, corresponding to the number of conditions in our dataset. The bars indicate the number of modules. (TIFF) [file pgen.1011599.s004.tiff]

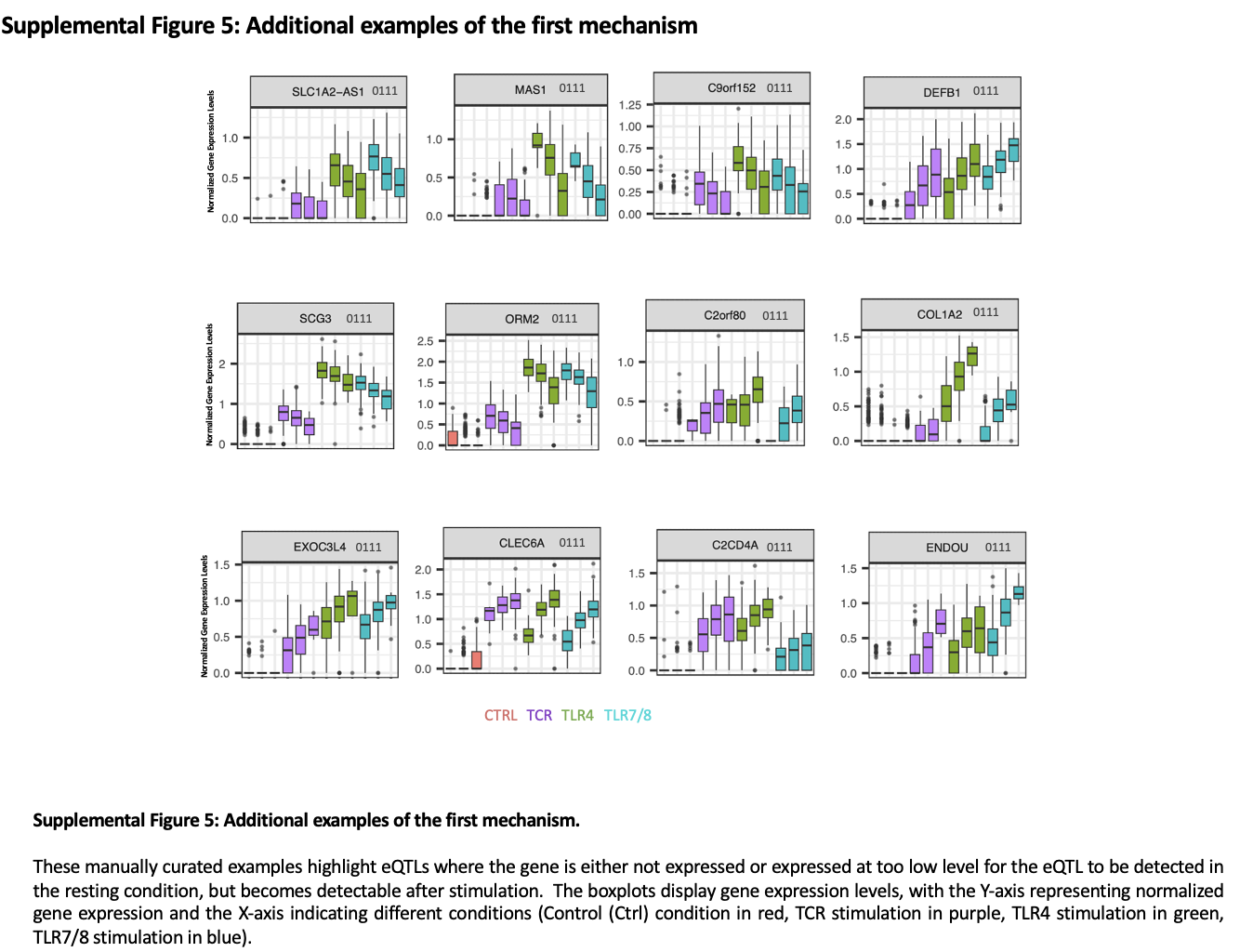

Supplement: S5 Fig — These manually curated examples highlight eQTLs where the gene is either not expressed or expressed at too low level for the eQTL to be detected in the resting condition, but becomes detectable after stimulation. The boxplots display gene expression levels, with the Y-axis representing normalized gene expression and the X-axis indicating different conditions (Control (Ctrl) condition in red, TCR stimulation in purple, TLR4 stimulation in green, TLR7/8 stimulation in blue). (TIFF) [file pgen.1011599.s005.tiff]

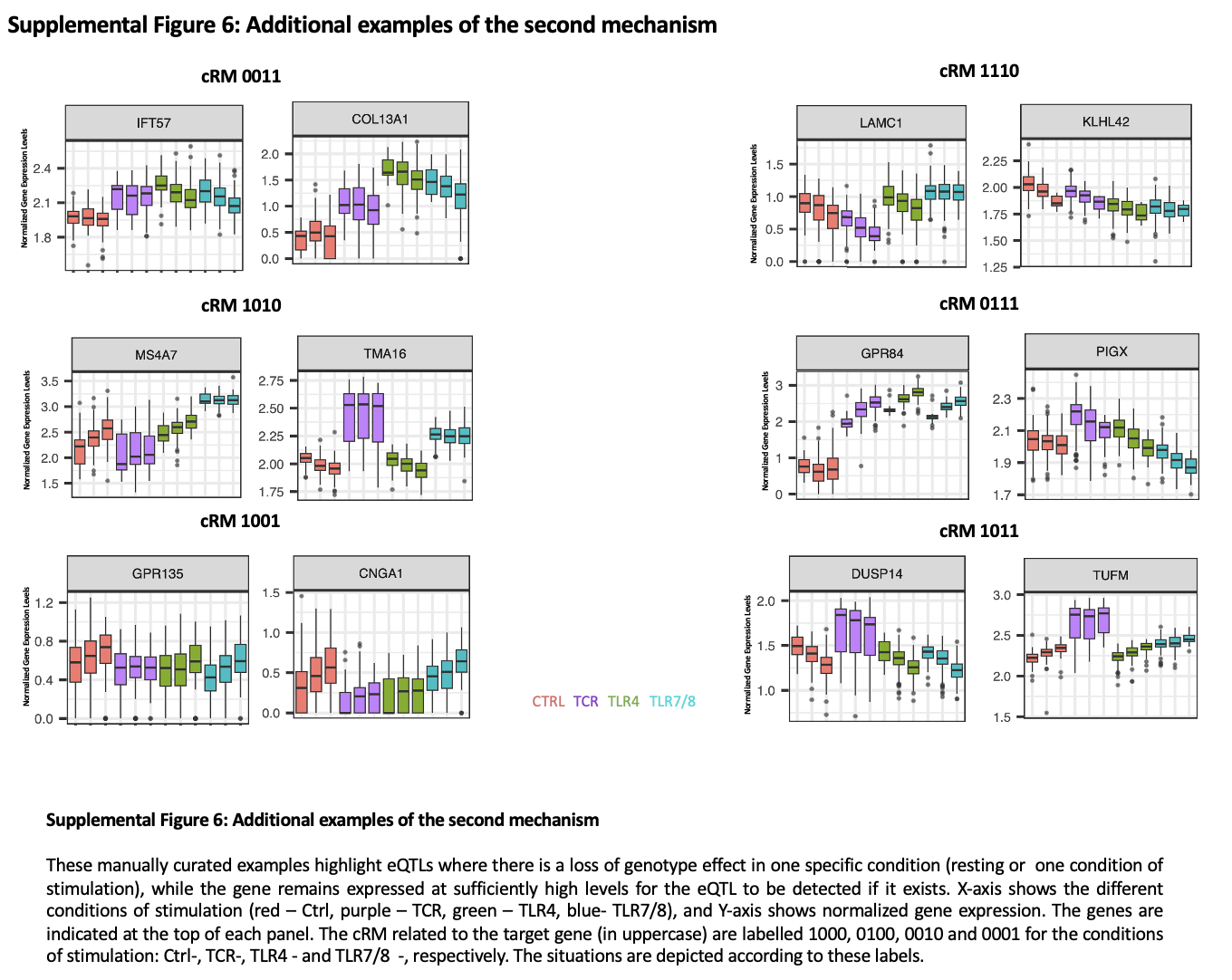

Supplement: S6 Fig — These manually curated examples highlight eQTLs where there is a loss of genotype effect in one specific condition (resting or one condition of stimulation), while the gene remains expressed at sufficiently high levels for the eQTL to be detected if it exists. X-axis shows the different conditions of stimulation (red – Crtl, purple – TCR, green – TLR4, blue- TLR7/8), and Y-axis shows normalized gene expression. The genes are indicated at the top of each panel. The cRM related to the target gene (in uppercase) are labelled 1000, 0100, 0010 and 0001 for the conditions of stimulation: Ctrl-, TCR-, TLR4 - and TLR7/8 -, respectively. The situations are depicted according to these labels. (TIFF) [file pgen.1011599.s006.tiff]

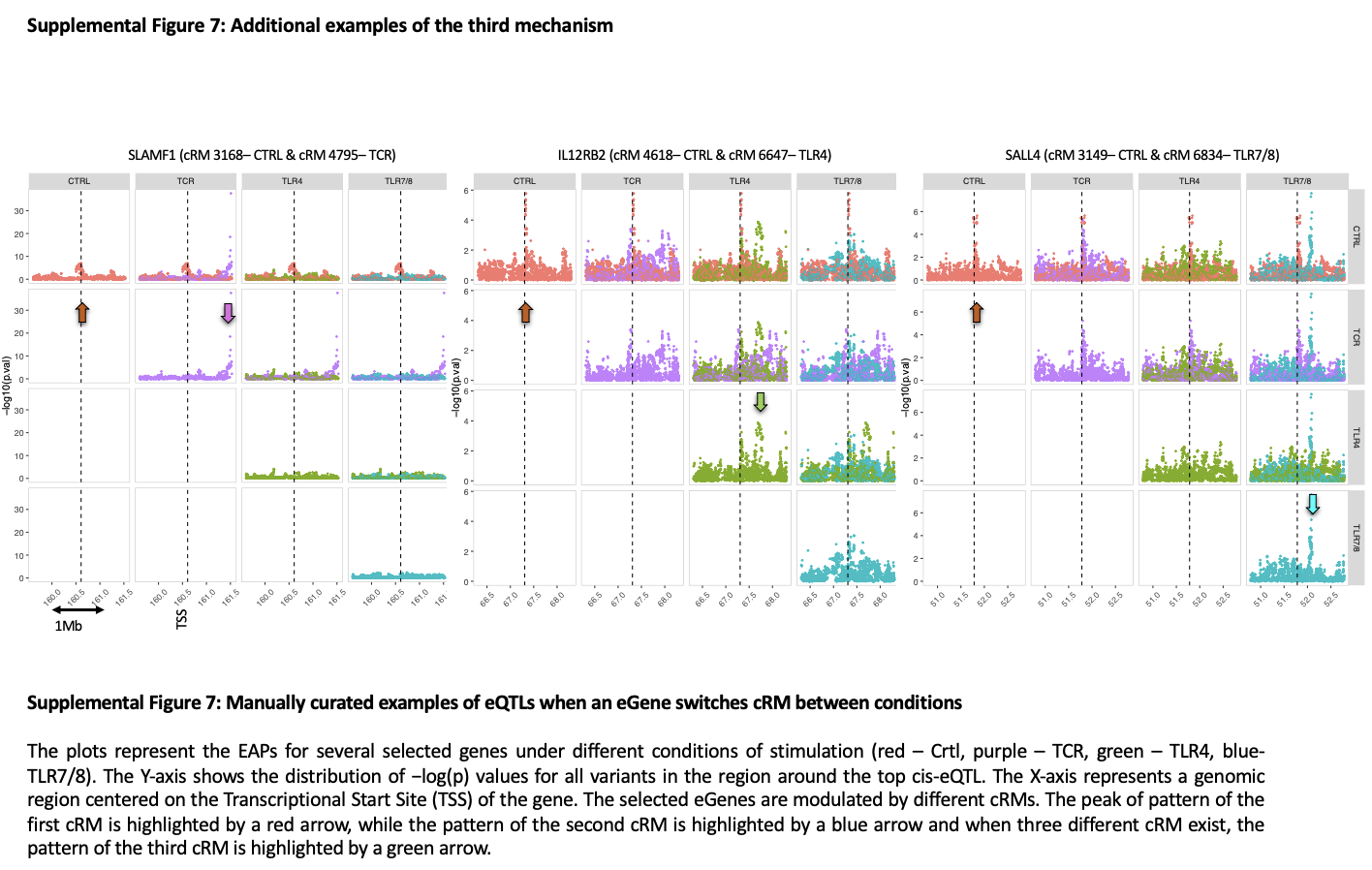

Supplement: S7 Fig — The plots represent the EAPs for several selected genes under different conditions of stimulation (red – Crtl, purple – TCR, green – TLR4, blue- TLR7/8). The Y-axis shows the distribution of −log(p) values for all variants in the region around the top cis-eQTL. The X-axis represents a genomic region centered on the Transcriptional Start Site (TSS) of the gene. The selected eGenes are modulated by different cRMs. The peak of pattern of the first cRM is highlighted by a red arrow, while the pattern of the second cRM is highlighted by a blue arrow and when three different cRM exist, the pattern of the third cRM is highlighted by a green arrow. (TIFF) [file pgen.1011599.s007.tiff]

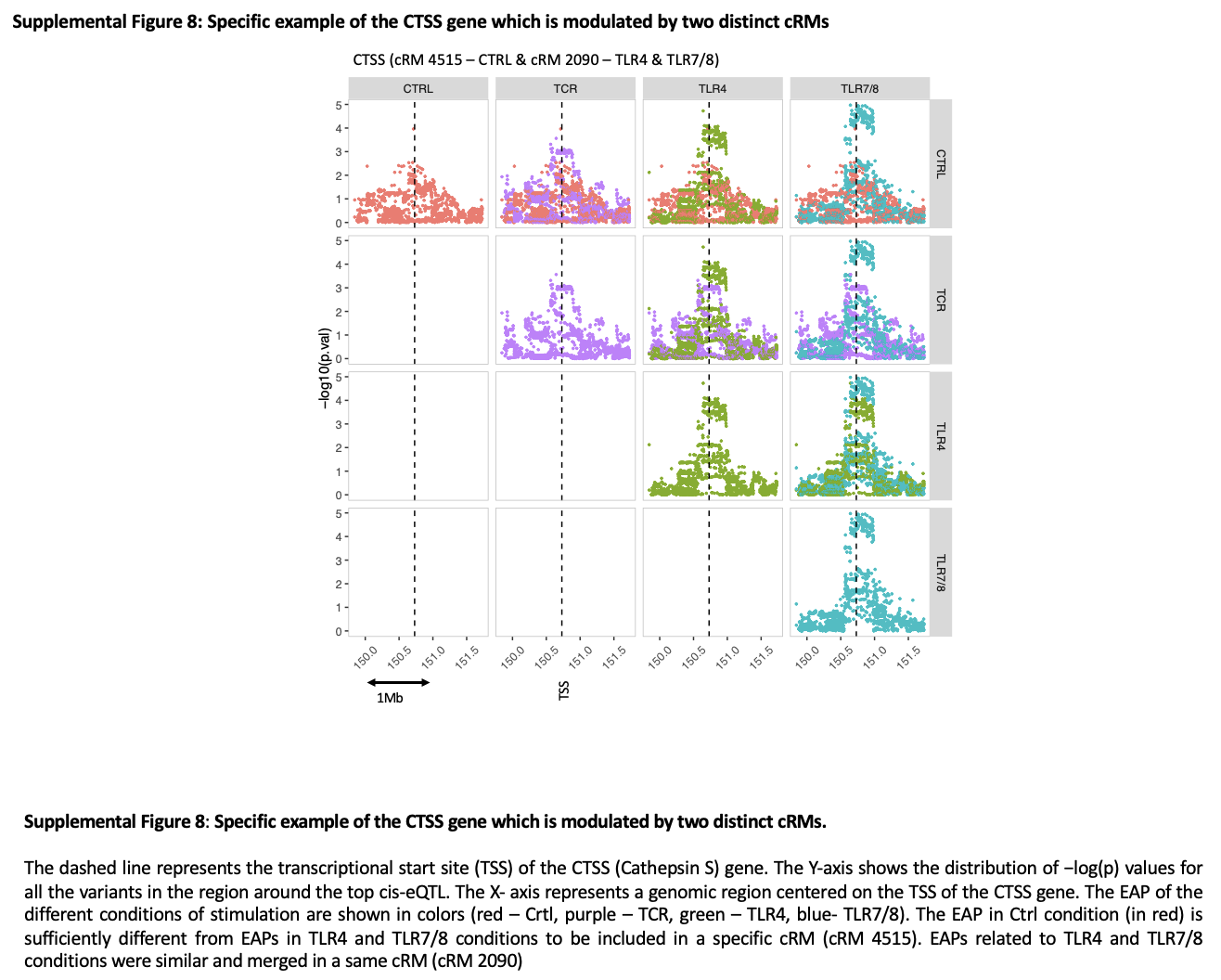

Supplement: S8 Fig — The dashed line represents the transcriptional start site (TSS) of the CTSS (Cathepsin S) gene. The Y-axis shows the distribution of −log(p) values for all the variants in the region around the top cis-eQTL. The X- axis represents a genomic region centered on the TSS of the CTSS gene. The EAP of the different conditions of stimulation are shown in colors (red – Crtl, purple – TCR, green – TLR4, blue- TLR7/8). The EAP in Ctrl condition (in red) is sufficiently different from EAPs in TLR4 and TLR7/8 conditions to be included in a specific cRM (cRM 4515). EAPs related to TLR4 and TLR7/8 conditions were similar and merged in a same cRM (cRM 2090). (TIFF) [file pgen.1011599.s008.tiff]

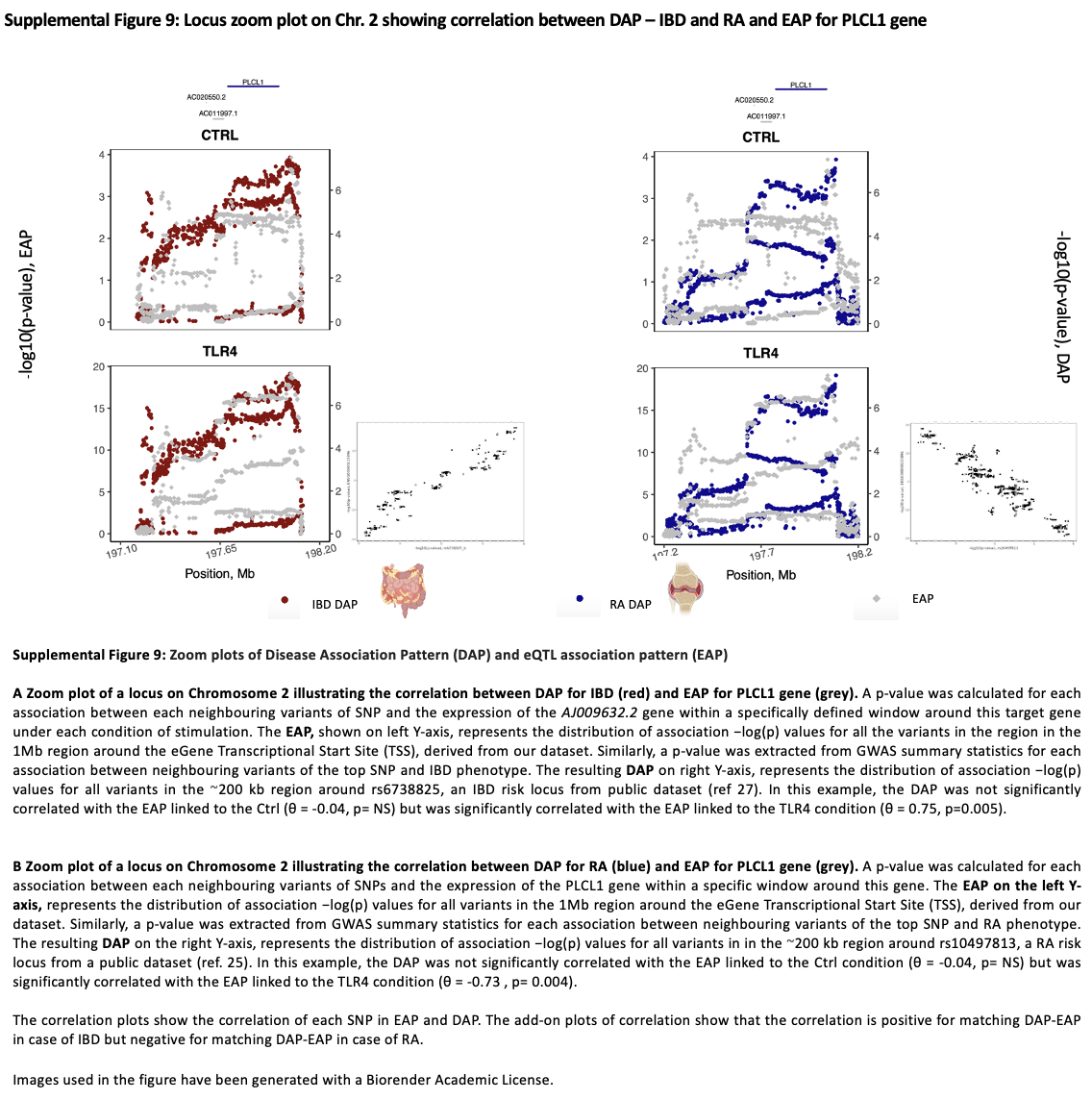

Supplement: S9 Fig — A Zoom plot of a locus on Chromosome 2 illustrating the correlation between DAP for IBD (red) and EAP for PLCL1 gene (grey). A p-value was calculated for each association between each neighbouring variants of SNP and the expression of the AJ009632.2 gene within a specifically defined window around this target gene under each condition of stimulation. The EAP, shown on left Y-axis, represents the distribution of association −log(p) values for all the variants in the region in the 1Mb region around the eGene Transcriptional Start Site (TSS), derived from our dataset. Similarly, a p-value was extracted from GWAS summary statistics for each association between neighbouring variants of the top SNP and IBD phenotype. The resulting DAP on right Y-axis, represents the distribution of association −log(p) values for all variants in the ~200 kb region around rs6738825, an IBD risk locus from public dataset (ref 27). In this example, the DAP was not significantly correlated with the EAP linked to the Ctrl (θ = -0.04, p= NS) but was significantly correlated with the EAP linked to the TLR4 condition (θ = 0.75, p=0.005). B Zoom plot of a locus on Chromosome 2 illustrating the correlation between DAP for RA (blue) and EAP for PLCL1 gene (grey). A p-value was calculated for each association between each neighbouring variants of SNPs and the expression of the PLCL1 gene within a specific window around this gene. The EAP on the left Y-axis, represents the distribution of association −log(p) values for all variants in the 1Mb region around the eGene Transcriptional Start Site (TSS), derived from our dataset. Similarly, a p-value was extracted from GWAS summary statistics for each association between neighbouring variants of the top SNP and RA phenotype. The resulting DAP on the right Y-axis, represents the distribution of association −log(p) values for all variants in in the ~200 kb region around rs10497813, a RA risk locus from a public dataset (ref. 25). In this example, the DAP was [file pgen.1011599.s009.tiff]

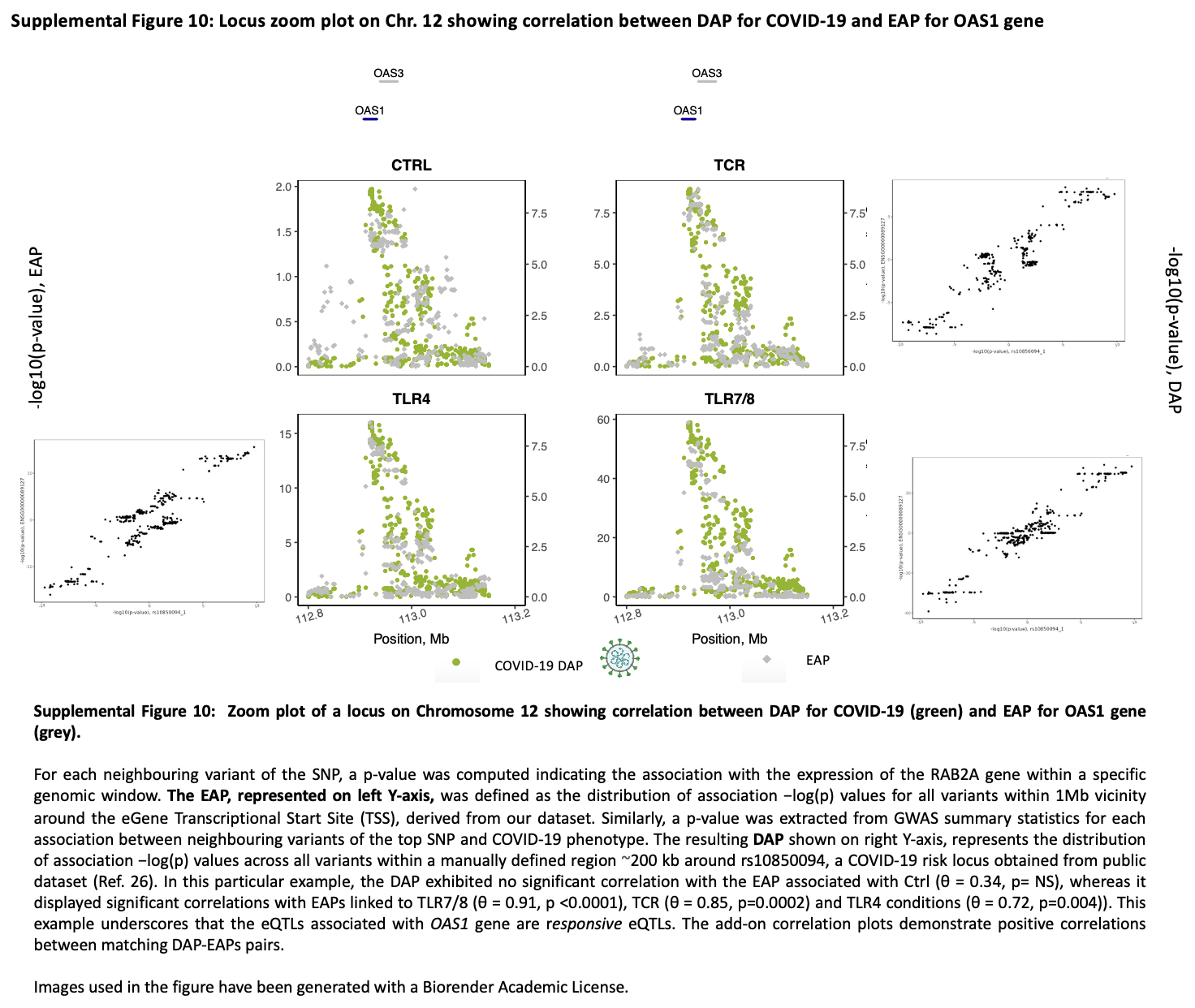

Supplement: S10 Fig — For each neighbouring variant of the SNP, a p-value was computed indicating the association with the expression of the RAB2A gene within a specific genomic window. The EAP, represented on left Y-axis, was defined as the distribution of association −log(p) values for all variants within 1Mb vicinity around the eGene Transcriptional Start Site (TSS), derived from our dataset. Similarly, a p-value was extracted from GWAS summary statistics for each association between neighbouring variants of the top SNP and COVID-19 phenotype. The resulting DAP shown on right Y-axis, represents the distribution of association −log(p) values across all variants within a manually defined region ~200 kb around rs10850094, a COVID-19 risk locus obtained from public dataset (Ref. 26). In this particular example, the DAP exhibited no significant correlation with the EAP associated with Ctrl (θ = 0.34, p= NS), whereas it displayed significant correlations with EAPs linked to TLR7/8 (θ = 0.91, p <0.0001), TCR (θ = 0.85, p=0.0002) and TLR4 conditions (θ = 0.72, p=0.004)). This example underscores that the eQTLs associated with OAS1 gene are responsive eQTLs. The add-on correlation plots demonstrate positive correlations between matching DAP-EAPs pairs. Images used in the figure have been generated with a Biorender Academic License. (TIFF) [file pgen.1011599.s010.tiff]
