# Supplementary material for: The identification of blood-derived response eQTLs reveals complex effects of regulatory variants on inflammatory and infectious disease risk
Source: PLoS Genet. 2025 Apr 10;21(4):e1011599. doi: 10.1371/journal.pgen.1011599 (PMC12013874; doi:10.1371/journal.pgen.1011599)
Supplement: S1 Data — (DOCX) [file pgen.1011599.s012.docx]

**The identification of blood-derived response eQTLs reveals complex effects of regulatory variants on inflammatory and infectious Disease risk**

**Supplementary material**

**Exploring the deconvolution of whole blood tissue and influence on eQTL discovery**

Whole blood contains multiple cell types. Hence, whole blood eQTL can either reflect genotype-dependent changes in steady state RNA levels within one or more cell types (scenario 1), or genotype-dependent changes in cell type proportions without changes in steady state RNA levels within cell types (scenario 2). GWAS performed in European cohorts identified 7122 loci influencing blood cell types (1). To determine whether some of our eQTLs might result from the second scenario, we first performed colocalization analyses between our eQTL catalogue and the corresponding loci. We focused on the 243 top risk loci influencing the abundance of four major cell types (lymphocytes, monocytes, eosinophils and neutrophils) (See methods). We identified 11 matching eQTL involving 6 genes in 6 risk loci (**Table 1**). All concerned eQTL modules but one (involving *PARVB* gene), were active in control conditions (i.e., prior to stimulation) as expected.

For changes in cell type proportions to underpin reQTL that are only detected after stimulation (i.e., absent in resting conditions), the stimulation would have to perturb cell-type proportions post-stimulation in a genotype-dependent manner (f.i. by affecting cell proliferation and/or survival). To test this, we estimated cell type proportions in all our samples using decon2 (2). We evaluated the correlation between FACS-measured (immunophenotyping) and decon2-estimated proportions in control samples for eight immune cell types. While the correlations varied across specific cell types, they were overall consistent with expectations as outlined in the original study (2) (**Table 2 and Fig 1**).

**
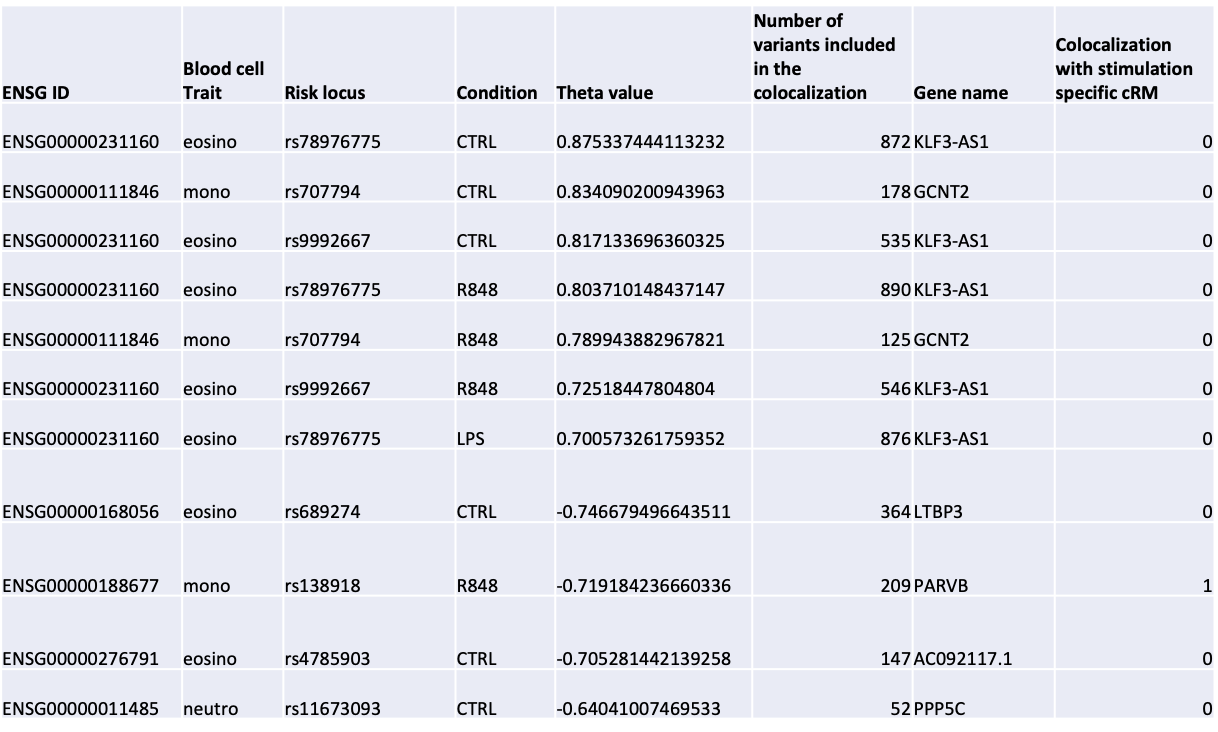
**

**Table 1: DAP-EAP correlations about Blood cell traits (summary statistics from Cell, 2020 Volume 182, Issue 5, 1214 - 1231.e11) (1)**

| **Cell line** | **Pearson coefficient** |
| --- | --- |
| CD19 B cells | 0.77 |
| CD3+ T cells | 0.30 |
| CD4+ T cells | 0.48 |
| CD8+ T cells | 0.52 |
| CD4- CD8- T cells | 0.31 |
| NK cells (CD3-CD56+) | 0.79 |
| Granulocytes | 0.67 |
| Monocytes | 0.58 |
| Lymphocytes | 0.48 |

**Table 2**: Proportions of nine cell lines analyzed at baseline using immunophenotyping. The known proportions of these cell lines were compared to the predicted proportions in control conditions. Decon2 was utilized for deconvolution of whole blood tissue.


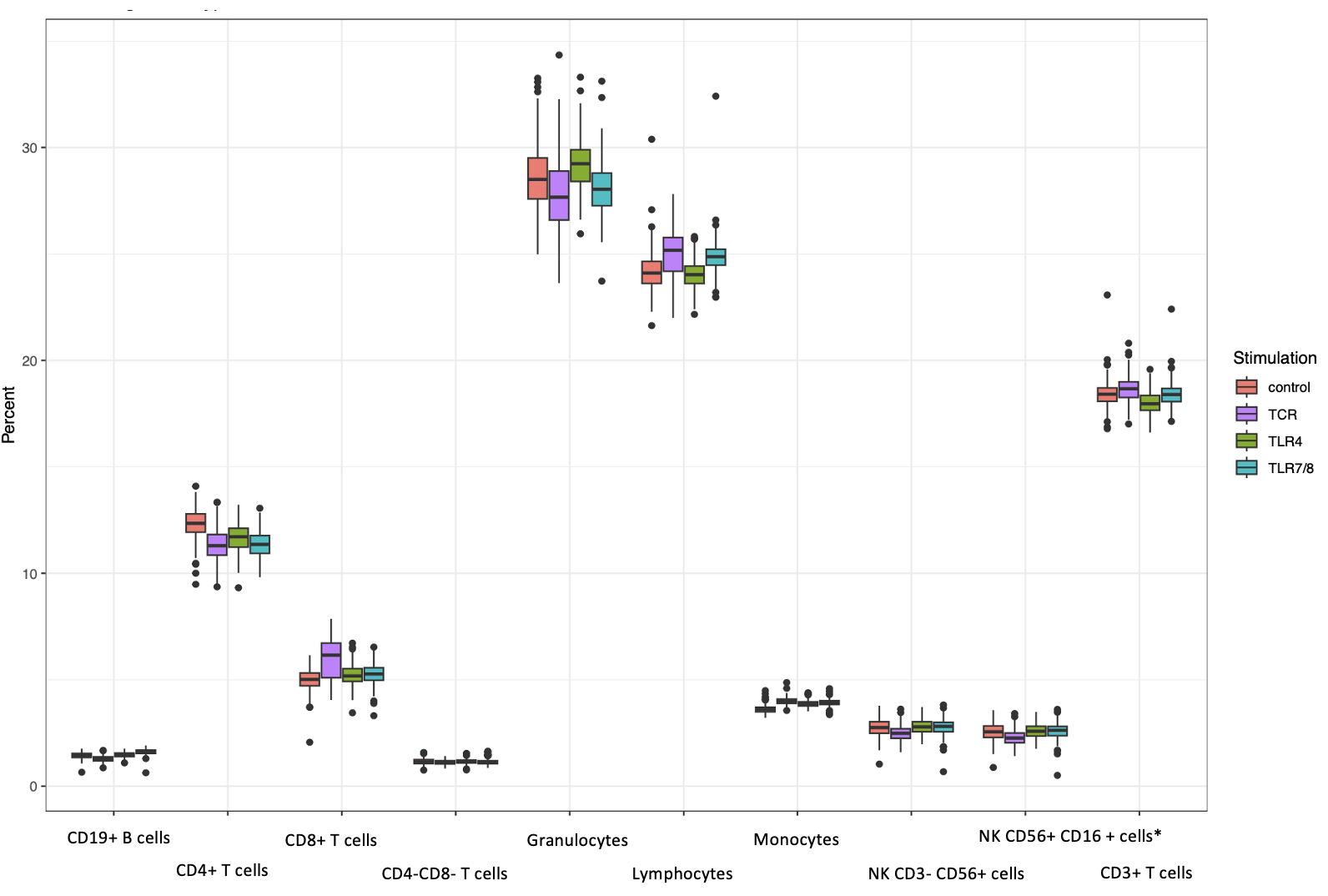


**Fig 1: Boxplots of Cell Line Proportions Across Four Conditions**: The boxplots present the proportions of cell lines across four conditions. ANOVA testing confirmed no significant differences between the conditions in each cell line. * indicates that the cell line proportion was not determined at baseline.

References

1. Vuckovic D, Bao EL, Akbari P, Lareau CA, Mousas A, Jiang T, et al. The Polygenic and Monogenic Basis of Blood Traits and Diseases. Cell. 2020;182(5):1214-31 e11.

2. Aguirre-Gamboa R, de Klein N, di Tommaso J, Claringbould A, van der Wijst MG, de Vries D, et al. Deconvolution of bulk blood eQTL effects into immune cell subpopulations. BMC Bioinformatics. 2020;21(1):243.
