# Supplementary material for: The identification of blood-derived response eQTLs reveals complex effects of regulatory variants on inflammatory and infectious disease risk
Source: PLoS Genet. 2025 Apr 10;21(4):e1011599. doi: 10.1371/journal.pgen.1011599 (PMC12013874; doi:10.1371/journal.pgen.1011599)
Supplement: Attachment — Submitted filename: Review - Point by point answers_final.pdf [file pgen.1011599.s014.pdf]

# Response to reviewers

## "The identification of blood-derived response eQTLs reveals complex effects of regulatory variants on inflammatory and infectious Disease risk"

By Claire Liefferinckx*, David Stern, Hélène Perée, Jérémie Bottieau, Alice Mayer, Christophe Dubussy, Eric Quertinmont, Vjola Tafciu, Charlotte Minsart, Vyacheslav Petrov, Alex Kvasz, Wouter Coppieters, Latifa Karim, Souad Rahmouni, Michel Georges, and Denis Franchimont

**Overall answer:**
We thank the reviewers and the editorial board for their positive and valuable feedback. In the following "response to reviewers" file, we have carefully addressed all remarks and comments. In complement, we have highlighted in yellow the related modifications carried out in the manuscript.

In light of these new changes, all authors believe that this updated, significantly improved version of the paper complies with the quality standards of the *Plos Genetics* journal, and we hope that it therefore qualifies for publication.

On behalf of all authors,

Claire Liefferinckx

Reviewer #1: Overview: Liefferinckx et al. performed cis-eQTL mapping using whole blood samples from N=406 healthy participants across four conditions, including three different immune stimulation and baseline resting. Briefly, the authors discovered ~50% of eQTLs were exclusively found after stimulation treatment, i.e., response eQTLs, which highlighted that eQTL can be context-specific and potentially better explain disease risk. The authors performed colocalization analysis between conditions and between genes to identify cis-regulatory modules and characterize condition specificity of eQTLs. Finally, the authors performed colocalization with GWAS immune traits and identified novel genes that are missed by using resting samples alone. This work will be likely of interest to the statistical genetics and human inflammatory disease communities. This manuscript is written well, and results presented clearly. Overall, I suggest providing more details in describing methods. With that said I have several comments.

Major Comments:

**1. For the results regarding association between rs1801274 and PC1-TCR, can the author provide more details in interpreting this finding? Given the PCs capture linear combinations of gene expression profile between individuals, can we interpret this as "trans-eQTL" effect in TCR condition? What's interesting is that rs1801274 is missense variant for FCGR2A, but this gene was not found to be correlated with PC1-TCR (Supplemental Table 2). In fact, after running PCA on the gene expression matrix within each condition, the authors can look at the loading matrix output to determine which genes have highest loading (positive or negative) on the top PC, which reflect correlations.**

We thank the reviewer for their insightful comments regarding rs1801274 and its association with PC1-TCR.

To clarify, there is a distinction between PC1 and PC1-TCR mentioned in the manuscript and Supplementary Table 2. PC1 refers to the principal component computed for the entire dataset. For the specific purpose of associating PCs as new phenotypes in a GWAS, PCs were recalculated for each paired condition (control-stimulated condition) to optimize the association signal (which is already detected by using PC1). We have taken care to explicitly communicate this distinction throughout the manuscript, figures, and legends to avoid any potential misunderstandings.

Indeed, the association between rs1801274 and PC1-TCR can be interpreted as a trans-eQTL effect. Principal components (PCs), such as PC1, capture linear combinations of gene expression profiles, and the loading values indicate which genes contribute the most (positively or negatively) to a given PC. To confirm the trans-eQTL nature of rs1801274, we conducted a dedicated trans-eQTL analysis using QTL tools, focusing specifically on rs1801274. We observed that a large number of genes driven by PC1 are linked to rs1801274 by trans-eQTL analyses (313/398).
We have now added the following to address this:

- Two new columns in Supplementary Table 2 presenting the detailed results of the trans-eQTL analysis for all matched genes in PC1.

- Further descriptions were given in the manuscript to clarify the methodology used for the trans-eQTL analysis.

Furthermore, while FCGR2A is a missense variant associated with rs1801274, we did not observe this gene in PC1 (Supplementary Table 2). An explanation could be that the SNP is a missense variant within the gene.
We have also included a brief explanation emphasizing that part of the TCR stimulation response in whole blood cell cultures might be mediated by the engagement of the FcγRIIA, expressed on monocytes/macrophages, neutrophils and dendritic cells, by circulating IgG -containing immune complexes or released IgG immunoglobulins (including the co-stimulatory signal of the soluble anti-CD28 antibody). This underscores the role of monocytes/macrophages and granulocytes in the elicitation and perpetuation of the TCR stimulation thanks to the ex vivo model/assay of whole blood cell cultures.

As asked by the reviewer, we have now included the loading values for the genes contributing to PC1 in Supplementary Table 2. These values highlight the genes most correlated with PC1, providing more context for understanding the association.

We hope these additions clarify and strengthen the interpretation of this finding. The changes have been highlighted in yellow in the revised manuscript.

**2. It would be helpful for the author provides more explanation on how the eQTL merging method works. How is it different from colocalization analysis described in Wallace C. PLoS Genet. 2021? Does multiple cRM for a given gene means multiple causal eQTL for this gene?**

We thank the reviewer for this insightful question. To address the query, we have clarified in the discussion how our eQTL merging method works, its distinctions from Bayesian colocalization approaches—specifically the one described by Wallace. in PLoS Genetics 2021—and how multiple cis-Regulatory Modules (cRMs) for a given gene relate to the presence of multiple causal eQTLs.

To address this, we have added a new paragraph in the Discussion section:

We employed the previously described θ-based approach (6) for colocalization analysis. This method compares vectors of log(1/p) values (referred to as "association patterns" or APs) in a specific chromosomal region for pairs of traits under investigation. These pairs could involve two eQTLs (EAP1 vs. EAP2) or an eQTL and a disease (EAP vs. DAP). Importantly, the p-value vectors do not need to originate from the same cohort, as long as both cohorts represent the same population and thus share a similar LD structure. The comparison metric is Pearson’s correlation coefficient, yet, (i) restricting analysis to SNPs with an association p-value < 0.05 for at least one trait, (ii) assigning greater weight to SNPs with stronger associations (“peaks” in the AP), (iii) incorporating the coherence of allelic effect signs across SNPs within traits, and (iv) providing a signed θ value (positive or negative) to indicate, for example, whether increased gene expression correlates with increased (positive θ) or decreased (negative θ) disease risk. The statistical significance of θ is assessed by comparing the observed θ value with those generated through permutation (randomizing phenotype-genotype pairs) for one of the traits. Permutations are performed for both traits, and the final p-value is the average

of the two empirical p-values. This approach accounts for variable LD patterns across the genome.
The θ-based approach is very similar to the SMR approach (49), with which it has been compared, and shown to provide comparable results (6). In essence, the SMR approach performs a correlation analysis between the effect sizes of the SNPs (β) rather than their log(1/p)-values. Neither method aims to fine-map association patterns or resolve whether signals arise from one or multiple causative variants (allelic heterogeneity). For both approaches, concluding that two APs match, requires the same set of causative variants to drive both signals. This set of causative variants, of unknown size, is referred to as a regulatory module (RM). Importantly, two APs influenced by distinct but overlapping sets of regulatory variants will not be deemed as "matching." In this regard, both methods differ from other colocalization methods, including coloc (50-52) and eCAVIAR (53). For example, coloc can exploit fine-mapping results (obtained f.i. using SuSiE (54)) and test for colocalization between pairs of multiple independent signals found to affect traits 1 and 2, respectively.

**3. In line with above, can the author provide interpretation on multigenic cRM? I am not surprised they are less common than monogenic cRMs given two genes have to be close enough to share the cis-eQTL association pattern. But it's not clear how to interpret this in the context of reQTLs across conditions.**

We thank the reviewer for this thoughtful remark, which highlights the need for further clarification on multigenic cis-Regulatory Modules (cRMs), particularly in the context of reQTLs across conditions.
We agree that multigenic cRMs are less common than monogenic cRMs, primarily due to the nature of cis-eQTL analysis, which requires multiple genes to be in close genomic proximity to share a common cis-regulatory variant. This physical constraint makes the occurrence of multigenic cRMs inherently less frequent, which aligns with our observations (453 multigenic cRMs vs. 7270 monogenic cRMs).

Among multigenic cRMs (453), the majority (353) includes modules with both baseline and reQTLs while 92 modules are response-specific QTLs (20%). Among monogenic cRMs (7270), the proportion of response-specific QTLs modules is significantly higher (4204/7270).

To illustrate this, we provide the following contingency table:

| Type | Multigenic cRMs | Monogenic cRMs |
|---|---|---|
| **Baseline and response eQTLs modules** | 353 | 1357 |
| **Specific baseline eQTLs modules** | 8 | 1709 |
| **Specific Response eQTLs modules** | 92 | 4204 |

We have incorporated these recalculations and interpretations into the revised manuscript for clarity.

**4. Regarding the three mechanisms to explain condition-specific cRM, can the author provide some mechanistic hypothesis? For example, could the treatment cause epigenetic**

**markers changes such as h3k27ac for enhancers and h3k4me3 for promoters, or chromatin change (open to close or vice versa), such that detection of eQTLs can be specific to certain condition or the sign of eQTL can differ between conditions.**

We thank the reviewer for this question, which provides an opportunity to explore mechanistic perspectives on the condition-specific cRMs observed in our study.

To address this, we have added a new paragraph in the Discussion section:

As we identified three mechanisms underlying condition-specific cRMs, mechanistic hypotheses may help elucidate how immune stimulation triggers these genetic effects. Previous studies have shown that eQTLs frequently overlap with active cis-regulatory elements, such as enhancers and promoters, which interact with target gene promoters across various immune cell types (46). Epigenetic modifications, including H3K27ac at enhancers and H3K4me3 at promoters, could drive chromatin accessibility changes, thereby influencing gene expression in a condition-specific manner. For example, LPS exposure has been shown to induce immune tolerance in monocytes through epigenetic modifications detectable as early as 1 hour post-stimulation (47). Similarly, recent studies on chromatin accessibility in lymphoblastoid cell lines revealed that chromatin accessibility quantitative trait loci (caQTLs) can also account for immune-mediated disease associations. These findings highlight the critical role of chromatin accessibility variations in shaping the regulatory landscape that modulates gene expression (48). These findings suggest that chromatin accessibility and histone modification dynamics could underlie the condition-specific activation of cRMs, potentially altering the detection or directionality of eQTLs under different conditions

**Minor Comments:**

**1. Figure 1A shows the separated clusters of four conditions, consistent with the expectation. To rule out the possibility that this may reflect batch effect, can the authors describe how RNA sequencing was done for these samples? Are they randomly assigned to different batches?**

This is an important point in RNA-sequencing management, we thank the reviewer to highlight this point. Indeed, to avoid batch effect by condition, we processed the samples per subject by mixing control and stimulated conditions. By doing so, the different batches of sequencing included all the conditions.
We added a sentence in methods to explain this point.

**2. Are the axis for Supplemental Figure 1c correctly labeled?**

Thank you for your sharp eye, we corrected this error.

**3. For Figure 2B, please add units for values shown on the heatmap Figure 2B. Is it 0-100%? Please switch the color scale to 0 as white and 100 as dark blue, it's very misleading right now. The color code doesn't match the captions describing this figure. For example, control is in red (stated as green). Please make sure the color code is correctly described.**

We thank the reviewer for highlighting this point, which brought attention to an error in the legend that made the figure difficult to interpret. The figure is not a heatmap of gene expression but rather a heatmap showing the correlation patterns of the 500 most variable genes. We have revised both the legend and the figure to improve clarity and readability.

**4. For Figure 3B, please choose 4 other different colors if you want to color it (not the same colors you used for four conditions), although I don't think it's necessary to color it as the upset plot is self-evident.**

We changed the colors to avoid confusion with the conditions.

**5. For Figure 3cdf, the x-axis looks like the allele count for risk allele, please annotate and describe the x-axis. Please label the units for y-axis.**

We labelled the y-axis in the figure and annoted the x-axis in the legend.

**6. Add label b in Figure 4.**

We correctly labelled the Figure 4

**7. The reference number in line 458 for EAP method looks like wrong. Should be (6) not (7).**

Thank you for your sharp eye, we corrected this error

**Reviewer #2:** Liefferinckx et al. presented their work on identifying cis-eQTLs that potentially regulate the expression of nearby genes in both resting and stimulated whole blood samples collected from a homogeneous group of healthy individuals. They identified response eQTLs specific to particular conditions and further provided three different scenarios to explain why a response eQTL was observed under stimulation. Lastly, they overlapped the eQTLs with publicly GWAS data on immune related diseases to identify candidate genes.

My main concerns are as follows:

**The authors used whole blood samples rather than sorted specific immune cell populations, such as dendritic cells (reference 8) and monocytes (references 8 & 11) for the innate immune response or T cells (reference 9) for adaptive immune response, as in previous work. Given that differences in abundances of specific immune cells and other blood cell traits across individuals have a genetic basis (Vuckovic et al 2020: https://doi.org/10.1016/j.cell.2020.08.008), could the eQTL signals that were identified in whole blood be confounded by blood cell traits? How would it potentially affect the identification of response eQTLs? I understand that performing cell sorting is not feasible at this stage, but have the authors considered using gene expression signatures to infer cell type composition, and assess its effects on the identification of (r)eQTLs? In addition, the authors should discuss the specific advantages and constraints of using whole blood in lines**

**289-290.**

We thank the reviewer for this important question and for highlighting the potential influence of blood cell traits on our eQTL and response eQTL (reQTL) analyses. We acknowledge that the use of whole blood presents both strengths and limitations, which we now discuss in more detail in the manuscript.

We address your question in a two-step approach.

First, we addressed the potential confounding of eQTL signals by Blood Cell Traits.
To investigate whether genetic variants associated with blood cell traits could confound our eQTL and reQTL results, we utilized publicly available GWAS meta-analysis data for blood cell traits (doi.org/10.1016/j.cell.2020.08.008).

For each genetic variant independently associated with a key blood cell phenotype (eosinophils, neutrophils, lymphocytes, and monocytes), we manually examined the distribution of association −log(p) values to define a comparison window, corresponding to a defined DAP. After validating the DAP window, we computed DAP-EAP correlations (as described previously for EAP-EAP comparisons) to systematically evaluate the overlap between blood cell trait-associated variants and our eQTL signals. We founded that a small part of blood cell traits colocalize with eQTLs in our dataset.

Second, we explored in Silico Deconvolution of Cell Composition
We applied an in silico deconvolution approach to estimate immune cell-type composition in whole blood using the Decon2 methodology (doi.org/10.1186/s12859-020-03576-5). This method was particularly relevant as it was developed using a cohort highly comparable to our GEOCODE cohort.

These new analyses have been included in a **Supplementary Materials**, as incorporating such results directly into the main text could disrupt the overall flow and clarity of the manuscript

Additionally, we conducted other analyses, which are outlined below. However, these results were not included in the manuscript (even in supplementary material) as they extend beyond the original scope of the study. For the reviewer's interest, we present these additional findings here:

We performed GWAS for the abundance of 9 cell types in unstimulated samples revealed one genome-wide significant hit for monocyte abundance on chromosome 13 (SNP id, log(1/p) ). However, this did not clearly match any of the signals reported in Vuckovic et al 2020, suggesting that it is a false positive.
We then performed GWAS using decon2-estimated cell type proportions in stimulated samples, corrected for decon2-estimated cell type proportions in the corresponding controls samples (to evaluate genotypic effects on changes in cell type proportions). We obtained one experiment-wide significant signal on chromosome 1, and one other genome-wide significant signal on chromosome 9. The chromosome 1 signal corresponded to the rs1801274 SNP influencing response after anti-CD3/antiCD28 stimulation reported in the first paragraph of the results section. However, as 122 of the 398 genes with expression level correlated to

transcriptome PC1 (also mapped to rs1801274, see SFig 1), were also part of the list of genes used by decon2 to infer cell type proportions, we cannot conclude at this point whether this QTL is mediated in whole or in part by chances in cell type proportions (rather than within cell type changes in gene expression level). Further work, that is beyond the scope of this study, is needed to reach definitive conclusions. The chromosome 9 signal corresponded to an increase in the proportion of granulocytes with increasing dosage of the T allele at the chr9:13557421:C:T SNP (rs 1324182)(Figures here below).
We mined our eQTL catalogue for EAP that would match the corresponding “granulocyte QTL” but did not find any.

GWAS on Delta cell proportion (LPS-CTL)

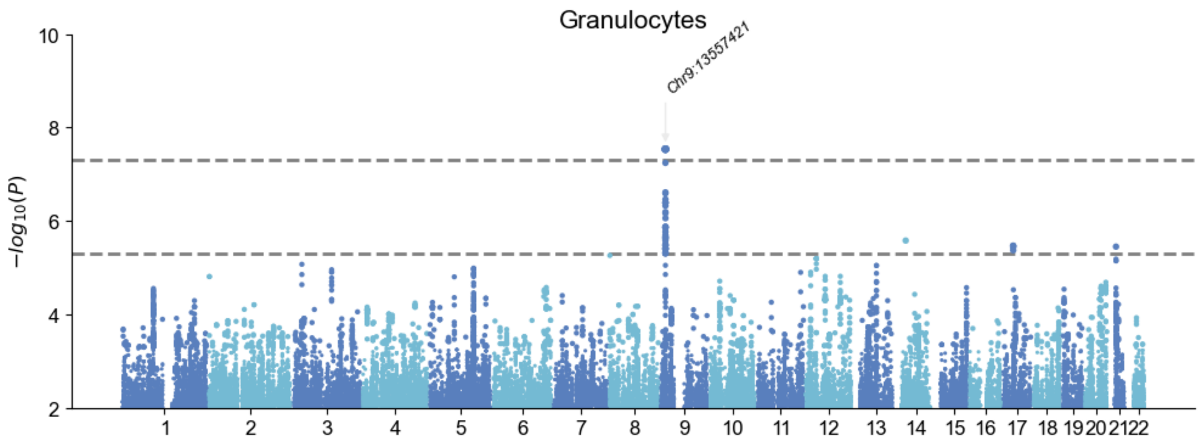


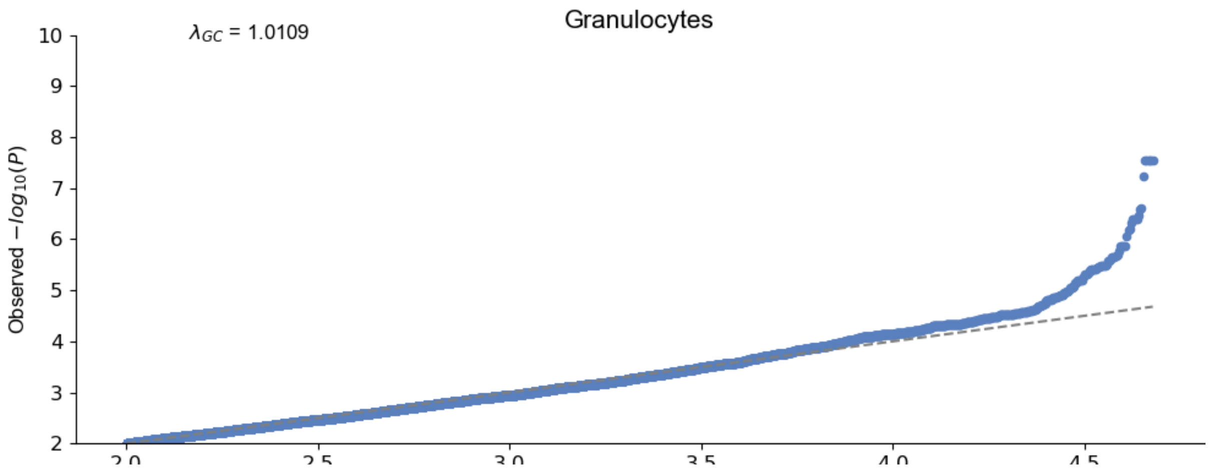

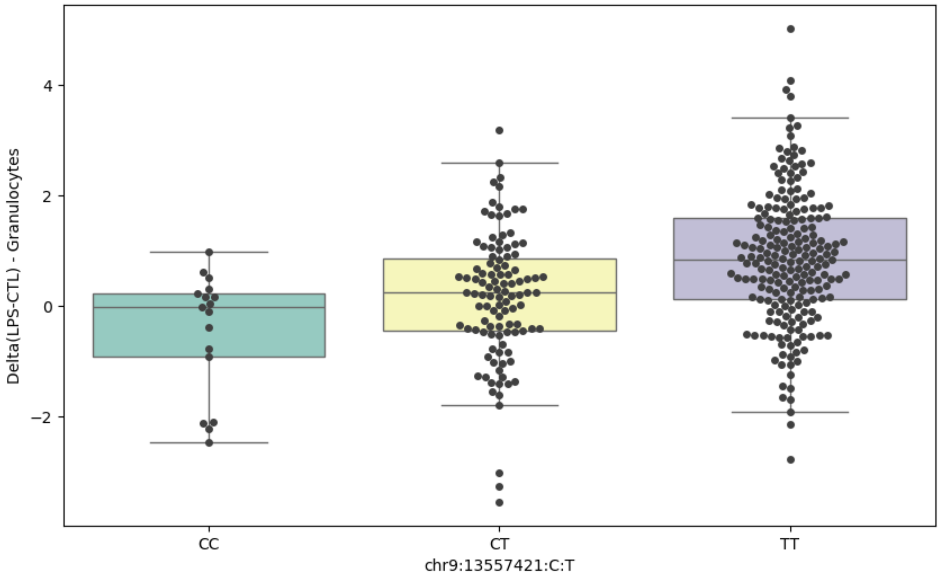


**Secondly, there is lack of methods for identifying response eQTLs. Are these defined simply as eQTLs that passed multiple testing correction in one condition but not in another, or are more formal statistical tests performed? The former is less ideal since an eQTL that showed suggestive evidence but did not pass multiple testing correction in other conditions (often due to limited power) could still be potentially shared across conditions, and thus not a true reQTL. Also, are directions of effects considered when defining reQTLs? For example, are eQTLs with opposite effect signs considered context specific eQTLs**?

We thank the reviewer for raising this important question, which is critical for ensuring that response eQTLs (reQTLs) identified in our study are indeed “true” response-specific eQTLs and not artifacts resulting from thresholding effects.

First, to address the concern that eQTLs passing multiple testing correction in one condition but not in another may still be shared across conditions (due to limited statistical power), we conducted additional analyses on a larger "extended dataset". This extended catalog includes 26,629 cis-eQTLs, allowing us to re-evaluate the condition-specificity of cRMs and reQTLs with increased statistical power. Importantly, the predominance of single condition-specific cRMs remained consistent in the extended dataset, supporting the robustness of our findings.

We have clarified this point in the Results section and provided further details on the generation and analysis of the extended dataset in the Methods section. Additionally, Supplementary Figure 4 now illustrates the distribution of cis-eQTLs across resting and stimulated conditions using this extended dataset.

Second, about the direction of effects in defining reQTLs, we agree that considering the direction of effects (positive vs. negative) is essential for defining reQTLs. In our analysis, we explicitly incorporated the sign of the effect sizes (sign of theta values).
For more clarity

- We added colors in Supplementary Figure 3 to highlight the directional effects of eQTLs, with blue indicating positive theta values and red indicating negative theta values.
- While most eQTLs retained consistent directional effects across conditions, we identified a few cRMs where the directions of effects differed. Specifically, Figure 3F shows rare cases where an eQTL had an opposite directional effect between resting and stimulated conditions.

Overall, based on this remark, we clarified the statistical framework for defining reQTLs, including the use of an extended dataset to ensure condition-specificity is not a result of limited power.
We explicitly addressed the inclusion of effect directionality in our analyses and provided clear examples to illustrate cases where effects were condition-specific or opposite.
We hope these clarifications address the reviewer's concerns and further support the robustness of our findings.

**Thirdly, the authors proposed three scenarios to explain context specific eQTL signals. It would be helpful to estimate the proportion that can be attributed to each of the three scenarios in this dataset, and to provide some comments on whether it can be generalisable to other cohorts/dataset.**

We thank the reviewer for this insightful comment, which enables a clearer understanding of the three mechanisms driving context-specific eQTL signals and their respective contributions. To estimate the proportion of context-specific eQTLs explained by each of the three proposed mechanisms, we adapted a methodology previously developed by our colleagues for a related study (doi.org/10.1101/2024.10.14.24315443). This approach allowed us to estimate and quantify the contributions of each mechanism within our dataset; However, a small variability has to be considered due to threshold of gene expression that we considered as well as the power calculation.

Our analysis confirms that this framework can be effectively applied to our study and suggests that it is generalizable to other cohorts and datasets. We have included these proportions in the revised manuscript to provide a more comprehensive interpretation of the observed mechanisms and their potential applicability across different biological contexts.

A new paragraph has also been added to the Methods section to describe the analyses in detail:

**"Investigating the Distribution of the Three Mechanisms Driving cRM Activity"**

Each monogenic cis-Regulatory Module (cRM) was characterized by indicating whether the gene is part of an eQTL (FDR <= 0.5) (1) or not (0) in the four conditions defined in order CTRL-TCR-LPS-R848. As an example, a gene matching as eQTL in the three conditions of stimulation but not in resting condition is labelled 0111 while another gene matching as eQTL in TCR and R848 conditions only is labelled 0101

We quantified the occurrence of changes in status for a gene regarding different conditions under specific scenarios.
First mechanism, a gene might be part of an eQTL in a condition but not in another due to a too low level of expression in the second condition. We calculated the number of cases where an eQTL transitioned from 0 to 1, corresponding to the first mechanism, where gene expression levels are too low in a condition for the eQTL to be detectable. Next, we assessed cases where a gene match as an eQTLs in several conditions but is splitted in multiple modules, representing the third mechanism, where a gene (eGene) switches cRMs between conditions, the genes is part of an eQTL in both conditions but the involved variants are distinct. The remaining activity patterns were attributed to the second mechanism, the gene is detected in both conditions at sufficient levels of expression but only match as an eQTL in one condition. This second mechanism was validated using a permutation-based methodology approach fully described, including the computational and statistical procedures, here: https://doi.org/10.1101/2024.10.14.24315443.

**Lastly, it appears that more eQTLs were identified in the resting samples only compared to stimulated samples (more control specific eQTLs as shown in Figure 3b and 3g; please provide specific numbers if it's not the case). Is this unexpected to the authors too? Given that control samples showed a high proportion of genes with low expression levels (figure 2B), one would expect that it leads to fewer eQTLs as expected under the first mechanism proposed by the authors.**

We thank the reviewer for raising this important question, which highlights a potential misinterpretation of Figures 3b and 3g. To address this, we have revised the figure legends to improve their clarity and readability. It is worth noting that Figures 3b and 3g depict the number of modules, but a single module may contain multiple eQTLs.
To provide a more precise answer to your question, we shall direct you to Supplementary Table 4, where we have detailed the number of samples and eQTLs detected per condition. This table better reflects the underlying data and provides clearer insights into the distribution of eQTLs across different conditions.

To clarify even more: our analysis identified a greater number of response eQTLs in stimulated conditions compared to baseline eQTLs. Specifically, we detected 3706 eQTLs under baseline conditions, whereas 9973 eQTLs were observed under stimulated conditions.
To further support this clarification, we included additional details in Supplementary Table 4, where we provided the number of detected genes per condition, demonstrating that the number of genes identified remains consistent across different conditions.

**Minor comments**:

**Discuss what's novel from this study compared to previous response eQTL studies.**

The novelties of our work compared to previous ones are based on several key aspects:

- We built a large cohort of 406 highly selected healthy individuals. The careful attention that we paid to the selection of subjects to minimize external influences on the immune response (and then response eQTLs); As reminder, this meticulous selection has been performed on a large cohort - this point has already been discussed.
- We report three mechanisms that may explain why an eQTL could only be revealed after immune stimulation
- We expanded the immune cis-regulome through their whole blood transcriptome obtained after stimulation with specific toll-like receptor (TLR) agonists and T-cell receptor (TCR) antagonist. Importantly, 72.9% of eQTLs, referred to as response eQTLs, were revealed through in vitro stimulation and would have been overlooked without stimulation
- We used a colocalization approach developed by our team. We have added a paragraph in discussion on this second aspect.
- We then mined this new catalogue of response (r)eQTLs, with public GWAS summary statistics of three diseases through a colocalization approach: inflammatory bowel diseases, rheumatoid arthritis and COVID-19 disease
- We identify reQTL-specific colocalizations for risk loci for which no matching eQTL were reported before, revealing interesting new candidate causal genes.
- we developed a browser to exploit our data (that reviewers can already use) https://tools.giga.uliege.be/cedar/publiclw

**Line 112: I don't think I can find how the driven genes were identified in Methods. Are these driven genes enriched in relevant pathways?**

We have added a new paragraph in Methods entitled "Transcriptomic analysis" where we have developed the methodology of normalization of gene expression counts (see other comment) and where we develop the analysis on PCA.
We performed "Reactom analysis" on these genes to highlight relevant pathways related to these genes. We added two sentences on this analysis in the main text and we added a Stable for Reactom results.

New paragraph added:

**"Transcriptomic analysis"**
The normalization of gene expression counts as well as the identification of differentially expressed genes were conducted using the DESeq2 v1.36.0 package, which accounts for library depth by calculating a normalization factor for each sample. The process involved the following steps: First, the geometric mean expression of each gene across all samples was computed to serve as a pseudo-reference. Second, the expression of each gene was divided by its pseudo-reference value to calculate ratios for each gene. The normalization factor for each sample was then determined as the median of these ratios specific to that sample. Finally, normalized counts were obtained by dividing the raw counts by the normalization

factor. Differentially expressed genes were identified normalizing each pair of comparison (Stimulation vs Control condition) together.
Principal Component Analysis (PCA) was applied taking the 500 most variable genes across the samples to confirm stratification in the dataset regarding the group stimulations. Principal components (PCs) were analyzed to identify the genes with the greatest contribution to each PC, based on their loading values, which indicate the influence of each gene on the PC. Greatest contributor genes were selected accordingly to an absolute value of correlation coefficient to PCs higher than 0.5.
Pathway enrichment analysis was performed on the top differentially expressed genes in PC1 using the public Reactome database. Only biological pathways with a false discovery rate (FDR) <0.05 were included. A heatmap was used to visualize the correlation patterns of the 500 most variable genes across subjects after 24 hours of stimulation.
.

**Line 458: please confirm if the correct reference is 6 or 7.**

Thank you for your sharp eye, we corrected this error.

**Line 424: please provide more details on the processing of the gene expression data. For instance, how the data were normalised, was the normalisation performed within each condition or across conditions, how correction for covariates was performed (before or after normalisation), etc**

This is a key point in the first step of RNA-sequencing data processing, we thank the reviewer to highlight this point. We added several sentences in methods to explain this point.

**Some work is needed to improve the readability:**
**• Supplementary Figure 1: what does the x-axis represent in panel C? The y-axis indicates PC1 but panel C looks identical to the TCR group in panel A; however, I think the authors calculated PCs in each condition separately for this GWAS. Could the authors please clarify?**

Regarding the labels of axis, there was an error that we corrected. About the supplementary Figure 1a and C, indeed the PCA are identical in terms of distribution of PC1 and PC2 but we changed two things:

1. We removed the groups control and groups TLR4 and TLR7/8 for improving graph visibility.
2. We flagged the dots from TCR group according the genotype (AA, AG, GG) of SNP rs1801274.

We slightly changed the legend of the supplementary Figure to avoid any misunderstanding.

- **Figure 2B showing expression heatmap: Could the authors explain what the scale from 0 to 100 means? LPS stimulation (TLR4) is green, not plink as stated in the current legend.**

We thank the reviewer for highlighting this point, which brought attention to an error in the legend that made the figure difficult to interpret. The figure is not a heatmap of gene expression but rather a heatmap showing the correlation patterns of the 500 most variable genes. We have revised both the legend and the figure to improve clarity and readability.

- **Could the authors make text in Figure 3 (especially b and g) bigger?**

We adapted the text of legends in Figure 3 to have them a lit bit bigger.

- **Figure 3E: the legend refers to a red arrow but I can't see any**

We adapted the Figure 3E for the red arrow be more visible

**Regarding data sharing: would the summary statistics for cis-eQTLs, response eQTLs, the merged cis regulatory module (cRM) that authors defined in this study, and colocalised or matched eQTL – GWAS signals be made publicly available? These data should not involve any sensitive information, and they would be valuable for future research**.

We fully agree with the reviewer. The process of data deposit is still pending but at the end of process on EGA archive platform (ID 1485 – GEOCODE Cohort) https://ega-archive.org/about/ega/

In addition, we developed a browser to exploit our data (that reviewers can already use) https://tools.giga.uliege.be/cedar/publiclw
